# Supplementary material for: Is Hearing Loss a Risk Factor for Idiopathic Parkinson’s Disease? An English Longitudinal Study of Ageing Analysis
Source: Brain Sci. 2023 Aug 12;13(8):1196. doi: 10.3390/brainsci13081196 (PMC10452744; doi:10.3390/brainsci13081196)
Supplement: Supplementary file 1 [file brainsci-13-01196-s001.zip › brainsci-2529550-supplementary.pdf]

Table S1. Change in self-reported hearing capabilities across waves.

**Self-reported hearing capabilities starting point**	**Change across waves**							
	Change from W1→W2 (% proportion)	Change from W2→W3 (% proportion)	Change from W3→W4 (% proportion)	Change from W4→W5 (% proportion)	Change from W5→W6 (% proportion)	Change from W6→W7 (% proportion)	Change from W7→W8 (% proportion)	Change from W8→W9 (% proportion)
1; Excellent	W1=1 → W2= 1 (18.2%)	W2=1 → W3= 1 (51.2%)	W3=1 → W4= 1 (52.8%)	W4=1 → W5= 1 (51.8%)	W5=1 → W6= 1 (52.2%)	W6=1 → W7= 1 (51.1%)	W7=1 → W 8= 1 (48.7%)	- *
	W1=1 → W2 = 2 (29.2%)	W2=1 → W3= 2 (30.9%)	W3=1 → W4= 2 (32.4%)	W4=1 → W5= 2 (34.8%)	W5=1 → W6= 2 (31.4%)	W6=1 → W7= 2 (34.1%)	W7=1 → W8= 2 (33.2%)	-*
	W1=1 → W2 = 3 (28.4%)	W2=1 → W3 = 3 (16.3%)	W3=1 → W4 = 3 (12.9%)	W4=1 → W5 = 3 (11.8%)	W5=1 → W6 = 3 (14.2%)	W6=1 → W7 = 3 (13.1%)	W7=1 → W8 = 3 (16%)	-*
	W1=1 → W2 = 4 (17.8%)	W2=1 → W3 = 4 (1.4%)	W3=1 → W4 = 4 (1.7%)	W4=1 → W5 = 4 (1.3%)	W5=1 → W6 = 4 (1.7%)	W6=1 → W7 = 4 (1.3%)	W7=1 → W8= 4 (1.4%)	-*
	W1=1 → W2 = 5 (6.4%)	W2=1 → W3 = 5 (0.2%)	W3=1 → W4 = 5 (0.2%)	W4=1 → W5 = 5 (0.3%)	W5=1 → W6 = 5 (0.4%)	W6=1 → W7 = 5 (0.4%)	W7=1 → W8 = 5 (0.7%)	-*
2; Very good	W1=2 → W2= 1 (21.6%)	W2=2 → W3= 1 (19.2%)	W3=2 → W4= 1 (18.8%)	W4=2 → W5= 1 (19.4%)	W5=2 → W6= 1 (18.5%)	W6=2 → W7= 1 (16.6%)	W7=2 → W8= 1 (18%)	W8=2 → W9= 1 (15.8%)
	W1=2 → W2 = 2 (27.8%)	W2=2 → W3 = 2 (41.3%)	W3=2 → W4 = 2 (47%)	W4=2 → W5 = 2 (47.3%)	W5=2 → W6 = 2 (44.4%)	W6=2 → W7 = 2 (46.7%)	W7=2 → W8 = 2 (46.2%)	W8=2 → W9 = 2 (45%)
	W1=2 → W2 = 3 (29.9%)	W2=2 → W3 = 3 (32.9%)	W3=2 → W4 = 3 (29.3%)	W4=2 → W5 = 3 (29.2%)	W5=2 → W6 = 3 (32%)	W6=2 → W7 = 3 (31.2%)	W7=2 → W8 = 3 (30.1%)	W8=2 → W9 = 3 (33.5%)
	W1=2 → W2 = 4 (16.2%)	W2=2 → W3 = 4 (6%)	W3=2 → W4 = 4 (4.1%)	W4=2 → W5 = 4 (3.9%)	W5=2 → W6 = 4 (4.3%)	W6=2 → W7 = 4 (4.7%)	W7=2 → W8 = 4 (5.3%)	W8=2 → W9 = 4 (5.2%)
	W1=2 → W2 = 5 (4.5%)	W2=2 → W3 = 5 (0.6%)	W3=2 → W4 = 5 (0.8%)	W4=2 → W5 = 5 (0.2%)	W5=2 → W6 = 5 (0.8%)	W6=2 → W7 = 5 (0.8%)	W7=2 → W8 = 5 (0.4%)	W8=2 → W9= 5 (0.5%)

3; Good	W1=3 → W2= 1 (19.5%)	W2=3 → W3= 1 (6.1%)	W3=3 → W4= 1 (6.9%)	W4=3 → W5= 1 (6.4%)	W5=3 → W6= 1 (5.8%)	W6=3 → W7= 1 (4.6%)	W7=3 → W8= 1 (4.3%)	W8=3 → W9= 1 (5.8%)
	W1=3 → W2 = 2 (27.8%)	W2=3 → W3 = 2 (20.7%)	W3=3 → W4 = 2 (24.6%)	W4=3 → W5 = 2 (23.1%)	W5=3 → W6 = 2 (22.8%)	W6=3 → W7 = 2 (22.4%)	W7=3 → W8 = 2 (22.3%)	W8=3 → W9 = 2 (21.1%)
	W1=3→ W2 = 3 (31.9%)	W2=3→ W3 = 3 (52%)	W3=3→ W4 = 3 (51.7%)	W4=3→ W5= 3 (51.6%)	W5=3→ W6= 3 (50.3%)	W6=3→ W7= 3 (53%)	W7=3→ W8= 3 (51.4%)	W8=3→ W9= 3 (53.2%)
	W1=3 → W2 = 4 (16.1%)	W2=3 → W3 = 4 (18.7%)	W3=3 → W4 = 4 (14.9%)	W4=3 → W5 = 4 (16.8%)	W5=3 → W6 = 4 (19%)	W6=3 → W7 = 4 (17.6%)	W7=3 → W8 = 4 (18.8%)	W8=3 → W9 = 4 (17.2%)
	W1=3 → W2 = 5 (4.7%)	W2=3 → W3 = 5 (2.5%)	W3=3 → W4 = 5 (1.9%)	W4=3 → W5 = 5 (2.1%)	W5=3 → W6 = 5 (2.1%)	W6=3 → W7 = 5 (2.4%)	W7=3 → W8 = 5 (3.2%)	W8=3 → W9 = 5 (2.7%)
4; Fair	W1=4→ W2= 1 (19.2%)	W2=4→ W3= 1 (1.2%)	W3=4→ W4= 1 (1.6%)	W4=4→ W5= 1 (1.2%)	W5=4→ W6= 1 (1.3%)	W6=4→ W7= 1 (1.6%)	W7=4→ W8= 1 (1.5%)	W8=4→ W9= 1 (2.2%)
	W1=4 → W2 = 2 (26.4%)	W2=4 → W3 = 2 (5.8%)	W3=4 → W4= 2 (6.5%)	W4=4 → W5= 2 (6.6%)	W5=4 → W6= 2 (7.3%)	W6=4 → W7= 2 (7.9%)	W7=4 → W8= 2 (6.1%)	W8=4 → W9= 2 (6.1%)
	W1=4→ W2 = 3 (31.9%)	W2=4→ W3= 3 (27.6%)	W3=4→ W4= 3 (31.7%)	W4=4→ W5= 3 (30.1%)	W5=4→ W6= 3 (27.3%)	W6=4→ W7= 3 (30.8%)	W7=4→ W8= 3 (31.1%)	W8=4→ W9= 3 (31.4%)
	W1=4 → W2 = 4 (17%)	W2=4 → W3 = 4 (53.2%)	W3=4 → W4 = 4 (50%)	W4=4 → W5 = 4 (50.7%)	W5=4 → W6 = 4 (51.1%)	W6=4 → W7 = 4 (49.2%)	W7=4 → W8 = 4 (49.5%)	W8=4 → W9 = 4 (49.1%)
	W1=4→ W2 = 5 (5.5%)	W2=4→ W3 = 5 (12.2%)	W3=4→ W4 = 5 (10.2%)	W4=4→ W5 = 5 (11.4%)	W5=4→ W6 = 5 (13%)	W6=4→ W7 = 5 (10.5%)	W7=4→ W8 = 5 (11.8%)	W8=4→ W9 = 5 (11.2%)
5; Poor	W1=5→ W2= 1 (19.3%)	W2=5→ W3= 1 (0.8%)	W3=5→ W4= 1 (0.6%)	W4=5→ W5= 1 (1.1%)	W5=5→ W6= 1 (0.8%)	W6=5→ W7= 1 (1.3%)	W7=5→ W8= 1 (0.7%)	W8=5→ W9= 1 (0.6%)
	W1=5 → W2 = 2 (29%)	W2=5 → W3 = 2 (3.6%)	W2=5 → W3 = 2 (2.6%)	W4=5 → W5 = 2 (3.6%)	W5=5 → W6 = 2 (2.5%)	W6=5 → W7 = 2 (3.5%)	W7=5 → W8 = 2 (5.4%)	W8=5 → W9 = 2 (4.1%)

W1=5→ W2 = 3 (30%)	W1=5→ W2 = 3 (15.8%)	W1=5→ W2 = 3 (16%)	W4=5→ W5 = 3 (12.4%)	W5=5→ W6 = 3 (13.7%)	W6=5→ W7 = 3 (16%)	W7=5→ W8= 3 (19.4%)	W8=5→ W9= 3 (16.7%)
W1=5 → W2 = 4 (16.6%)	W2=5 → W3 = 4 (33.1%)	W2=5 → W3 = 4 (35.3%)	W4=5 → W5 = 4 (32.9%)	W5=5 → W6 = 4 (35.2%)	W6=5 → W7 = 4 (30.1%)	W7=5 → W8 = 4 (27%)	W8=5 → W9 = 4 (30.8%)
W1=5→ W2 = 5 (5.1%)	W2=5→ W3= 5 (46.7%)	W2=5→ W3= 5 (45.5%)	W4=5→ W5= 5 (50%)	W5=5→ W6= 5 (47.8%)	W6=5→ W7= 5 (49.1%)	W7=5→ W8= 5 (47.5%)	W8=5→ W9= 5 (47.8%)

Note. Change in self-reported hearing capabilities is modelled from one wave to the next. Changes are reported as proportions e.g. the proportion of participants whose self-reported hearing capabilities at wave 1 were 1 (Excellent) and changed to 2 (Very good) at wave 2. Wx denotes the wave (i.e. W1= Wave 1, W2 = Wave 2, W3= Wave 3, W4= Wave 4, W5= Wave 5, W6-= Wave 6, W7= Wave 7, W8= Wave 8 and W9= Wave 9).

*There were no participants who met the study inclusion criterion within this parameter (i.e. there were no participants who had a self-reported hearing capabilities of 1 at wave 8, who did not have PD entering the study and had age data and self-reported hearing data available at wave 9) , therefore proportions could not be computed.
